# Immune Activation, Inflammation, and Non-AIDS Co-Morbidities in HIV-Infected Patients under Long-Term ART

**DOI:** 10.3390/v11030200

**Published:** 2019-02-27

**Authors:** Sonia Zicari, Libera Sessa, Nicola Cotugno, Alessandra Ruggiero, Elena Morrocchi, Carlo Concato, Salvatore Rocca, Paola Zangari, Emma C. Manno, Paolo Palma

**Affiliations:** 1 Research Unit in Congenital and Perinatal Infections, Academic Department of Pediatrics (DPUO), Division of Immunology and Infectious Diseases, Bambino Gesù Children’s Hospital, 00165 Rome, Italy; sonia.zicari@opbg.net (S.Z.); nicola.cotugno@opbg.net (N.C.); alessandra.ruggiero@opbg.net (A.R.); salvatore.rocca@opbg.net (S.R.); paola.zangari@opbg.net (P.Z.); emma.manno@opbg.net (E.C.M.); 2 Department of Systems Medicine, University of Rome Tor Vergata, 00133 Rome, Italy; libera.sessa@gmail.com (L.S.); elena.morrocchi@gmail.com (E.M.); 3 Department of Laboratories, Division of Virology, Bambino Gesù Children’s Hospital, 00165 Rome, Italy; carlo.concato@opbg.net

**Keywords:** inflammation, immune activation, premature aging, metabolic syndrome, HIV, ART, non-AIDS co-morbidities

## Abstract

Despite effective antiretroviral therapy (ART), people living with HIV (PLWH) still present persistent chronic immune activation and inflammation. This condition is the result of several factors including thymic dysfunction, persistent antigen stimulation due to low residual viremia, microbial translocation and dysbiosis, caused by the disruption of the gut mucosa, co-infections, and cumulative ART toxicity. All of these factors can create a vicious cycle that does not allow the full control of immune activation and inflammation, leading to an increased risk of developing non-AIDS co-morbidities such as metabolic syndrome and cardiovascular diseases. This review aims to provide an overview of the most recent data about HIV-associated inflammation and chronic immune exhaustion in PLWH under effective ART. Furthermore, we discuss new therapy approaches that are currently being tested to reduce the risk of developing inflammation, ART toxicity, and non-AIDS co-morbidities.

## 1. Introduction

Globally, 36.9 million people are living with the human immunodeficiency virus (HIV) [1]. Thanks to antiretroviral therapy (ART) introduction, people living with HIV (PLWH) have a longer life expectancy and decreased morbidity and mortality than untreated patients, but despite effective therapy the virus is not completely eradicated. Consequently, HIV is now considered a chronic disease, rather than a fatal one, in countries where ART is available [2]. The persistence of the virus supports a chronic status of immune activation and inflammation leading to the increased production of pro-inflammatory cytokines [3] and thymus dysfunction [4]. In addition, persistent inflammation exacerbates tissue damage in PLWH, particularly in the gastrointestinal tract, allowing microbial components to enter into circulatory system (microbial translocation), increasing inflammation [5]. This immunological status increases the risk for PLWH to develop non AIDS-related co-morbidities generally associated with immunosenescence, commonly observed in the elderly [6]. Furthermore, HIV infection and long-term ART have been associated with the development of metabolic syndrome (MetS) [7,8], which is a combination of metabolic disorders that includes hypertension, hyperglycemia, changes in fat distribution, increase of cholesterol low-density lipoprotein (LDL) and triglycerides, a and reduced level of cholesterol high-density lipoprotein (HDL) and may lead to cardiovascular diseases (CVDs) such as heart disease, stroke, and diabetes [8,9,10]. The goal of this review is to provide an overview of the most recent findings on the correlation between HIV and inflammation linked to immune activation and non-AIDS co-morbidities associated with metabolic complications in PLWH.

## 2. How HIV Infection Impacts Microbiota Composition, Chronic Inflammation, Immune Activation, and Premature Aging

Within a few weeks of HIV infection, the virus begins a massive assault on the gut, which undergoes a significant depletion of memory CD4+ T cells. Such depletion is followed by disruption of the tight junctions in the intestinal epithelium, which may not be fully restored even with early ART initiation (within six months of infection) [11,12]. The disruption of the gut leads to both an imbalance of the intestinal microbiota composition (dysbiosis) and to the release of bacterial products in the circulation (microbial translocation) that induce chronic immune activation and inflammation [5,13,14,15]. As regards dysbiosis, several studies demonstrated that, in comparison with healthy controls (HCs), PLWH present an altered microbiota composition with an increase of pro-inflammatory and potentially pathogenic bacteria and a decrease of beneficial ones [16,17,18]. Specifically, Larsen et al. demonstrated that some strains of *Prevotella*, abundant in the microbiota of PLWH, enhancing Th17-mediated mucosal inflammation, have a pro-inflammatory effect [19], while *Bacteroides*, which decrease in these patients, are capable of producing anti-inflammatory cytokines [20]. Indeed, Paquin-Proulx et al. demonstrated that some *Bacteroides* genera correlate with the production of invariant natural killer T cells in the gut, leading to a reduction of immune activation [21]. Furthermore, Vujkovic-Cvijin et al. found a positive correlation between *Prevotella* and immune activation, while *Bacteroides* abundance was negatively correlated with inflammatory markers in PLWH under ART or ART-naïve PLWH, highlighting their protective effect against inflammation [18].

**Figure 1:**
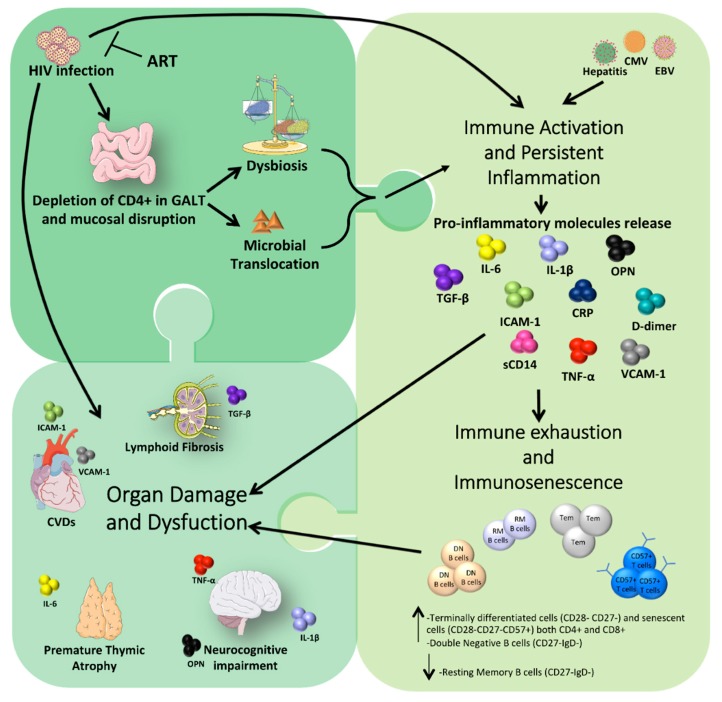
HIV infection causes both mucosal disruption and depletion of CD4+ T cells in gut-associated lymphoid tissue (GALT), altering the microbial composition (dysbiosis) and allowing microbial product to enter the circulatory system. Even with the introduction of antiretroviral therapy (ART), these two mechanisms lead to chronic immune activation and persistent inflammation that could also be enhanced by opportunistic co-infections. In turn, chronic activation and persistent inflammation result in (i) immune exhaustion and premature immune senescence, and in (ii) a direct damage of organs, through the release of pro-inflammatory cytokines. Images were obtained from Servier Medical Art images (http://smart.servier.com/).

As mentioned above, microbial translocation is another important cause of chronic inflammation. In particular, lipopolysaccharides (LPS), a component of the Gram-negative bacteria cell wall, is released from the leaky gut and is able to initiate a strong immune response. In fact, LPS binds CD14, found either in soluble form or anchored on the surface of monocytes and macrophages. The newly formed complex LPS/CD14 activates Toll-like receptor-4 (TLR4), leading to the production of pro-inflammatory cytokines [22,23,24,25]. Furthermore, this binding is also responsible for triggering the coagulation cascade, increasing the production of procoagulant tissue factors [6]. Indeed, it has been shown that soluble CD14 (sCD14) remains high in PLWH even with effective ART [26], and that it is associated with the risk of developing CVDs [27,28,29]. Overall, microbial translocation could be considered one of the major drivers of morbidity and mortality in HIV infection, since its role is to induce and sustain persistent inflammation [5,30,31,32]. As summarized in Figure 1, several mechanisms contribute to chronic inflammation in PLWH.

Chronic immune activation and persistent inflammation also affect the lymphoid tissue, leading to upregulation of transforming growth factor β (TGF-β), which stimulates collagen production. The collagen replaces the fibroblastic reticular network modifying the structure and function of lymphoid tissue with the progressive loss of naïve T cells [33,34,35]. As demonstrated by Sanchez et al., the introduction of ART did not reverse the lymphoid tissue fibrosis, maybe due to persistent inflammation and a low grade of virus replication [36].

Constant antigen stimulation produces other inflammatory biomarkers such as interleukin (IL)-6, IL-1β, tumor necrosis factor (TNF)-α, and C-reactive protein (CPR). Recently, it has been shown by Grund et al. that IL-6 and D-Dimer are independently associated with non-AIDS co-morbidities in PLWH, suggesting that treatment aiming to decrease these biomarkers may help to reduce morbidity and mortality in PLWH under ART [37]. It has also been shown that intercellular adhesion molecule (ICAM)-1, vascular cell adhesion molecule (VCAM)-1 and D-dimer, markers of CVDs [38,39,40], and monocyte chemotactic protein osteopontin (OPN), related to the risk of dementia, are elevated in PLWH [41].

The persistent inflammation also affects the functionality of the thymus, which is necessary for the achievement of complete immune recovery. Indeed, in untreated adults HIV infection causes chronic inflammation and immune activation that induce thymopoiesis, leading to long-term thymic dysfunction and clonal exhaustion of T cells [42]. Moreover, HIV-induced pro-inflammatory molecules sustain an abnormal development of regulatory T cells (Tregs) in the thymus, resulting in a lack of control of HIV and opportunistic pathogen infections [42]. Besides, thymic atrophy and fibrosis bring a decreased receptiveness to IL-7 that seems to be correlated to the continuous expression of type I interferons and decreased expression of IL-7Rα caused by IL-1β and IL-6, linked to cell death and thymopoiesis inhibition [43]. With the introduction of ART, thymic functionality is only partially rescued. Nonetheless, the early start of treatment in adults is necessary to avoid thymic dysfunction before the damage becomes irreversible [4]. In addition, the role of the thymus in maintaining efficient gut mucosal defense has been highlighted in several studies. De Voeght et al. found a link between thymic function failure, microbial translocation, and immune activation, suggesting that increasing thymic output can be used to correct aberrant activation caused by HIV infection or HIV-induced microbial translocation [44]. In line with this, Bourgeois et al., in a murine model of thymomectomy, show that the loss of Th17 cells causes an imbalance in host immunity, leading to gut barrier disruption, microbial translocation, and consequently, a persistent state of chronic immune activation [45].

The persistent inflammation is also sustained by co-infections with other viruses, such as hepatitis C virus (HCV), hepatitis B virus (HBV), cytomegalovirus (CMV), and Epstein-Barr virus (EBV). For example, Márquez et al. showed that HIV/HCV co-infected patients had higher levels of sCD14 and IL-6 as compared with those with chronic hepatitis or HIV-mono-infected individuals [46]. Moreover, recently, Maidji et al. demonstrated that CMV can disrupt epithelial junctions, leading to bacterial translocation and chronic inflammation in the gut. CMV-induced inflammation may attract HIV target cells, promoting HIV persistence [47]. In line with this, several studies showed an association between CMV infection and high levels of proviral HIV DNA [48,49,50]. Indeed, CMV can further foster HIV persistence, promoting inhibitory immune pathways (e.g., cellular exhaustion, programmed cell death protein (PD)-1, and IL-10 expression) and HIV-infected cell survival, thereby prompting the proliferation and clonal expansion of HIV-target cells and directly transactivating latent HIV [51,52].

HIV and immune activation persistence lead to premature aging of the immune system. In fact, several studies demonstrated that the immunological profiles of PLWH and elderly HCs show similarities [6,53,54,55]. In particular, it has been observed in both adults and pediatric patients that PLWH and HCs present an increased proportion of terminally differentiated cells (CD28−/CD27−) in CD4+ and CD8+ T cell compartments, expressing the typical marker of immunosenescence (CD57+) [56,57,58,59,60]. Another important marker of cellular senescence is telomere length shortening. Telomeres are essential for preserving genomic integrity and they become significantly shorter in the CD8+ T cells of PLWH compared with HCs [59].

Another similarity between PLWH and elderly HCs is the reduction of naïve T cells. As mentioned above, HIV leads to thymus dysfunction, which compromises the capacity to produce new T cells. Since the response to new antigens relies on the activation of naïve T cells, PLWH are unable to mount an effective immune response and, as in the elderly HC population, they have a reduced vaccine response [61,62].

## 3. Initiation and Duration of ART Can Affect Chronic Immune Activation and Inflammation

The timing of ART start is known to affect virological and immunological outcomes in HIV-infection: indeed, early treatment is associated with improved virus control and reduced levels of cellular virus reservoir [63,64]. One recent study by De Paula et al. showed that in HIV-infected adult patients, plasma levels of inflammatory biomarkers (IP-10, IL-18, sCD163) and CD8+CD38+HLA-DR+ T cell frequencies were significantly reduced after six months of ART in early-treated but not in late-treated individuals [65]. Furthermore, Allers et al. showed that in patients who initiated ART during acute HIV infection, mucosal CD4+ T cell numbers were well preserved, and markers of microbial translocation and inflammation reversed to normal [66]. On the other hand, this study showed that in PLWH, early ART improves CD4+ T cell differentiation but cannot prevent the persistent lack of total CD4+ T cells in mucosal tissue. In line with this latter study, Ghislain et al. showed that IL-6, sCD14, soluble tumor necrosis factor receptor 2 (sTNFR2), triglycerides, and insulin levels were also higher or tended to be higher in patients that received ART late after diagnosis than in those that received treatment sooner [67]. On the other hand, the impact of an early start to therapy on immunological recovery was debated by Amu et al., who showed that the expression of activation (HLA-DR and CD38 on CD4+ T cells) and terminal differentiation (CD127 on CD8+ T cells) markers in T cells were abnormal but comparable between early- and late-treated patients [68]. Similarly, Ruggiero et al. demonstrated that there was no difference in levels of soluble and cellular markers of activation measured in patients that started ART at different times after diagnosis [69,70]. These characteristics, alongside early treatment, minimize HIV reservoir size and diversity, prevent the quantitative loss of Th17 cells, and positively impact on local and systemic T cell activation [71,72,73,74,75].

Nonetheless, the demonstrated virological benefits of early therapy enable this strategy to be included in the current therapy recommendation both in adults and children, thereby promoting long life expectancy [76,77]. In the near future, long-ART treated PLWH will age facing the common consequences of immunosenescence compounded by additional health problems associated with infection and treatment toxicity.

## 4. Non-AIDS Co-Morbidities in PLWH

**Figure 2:**
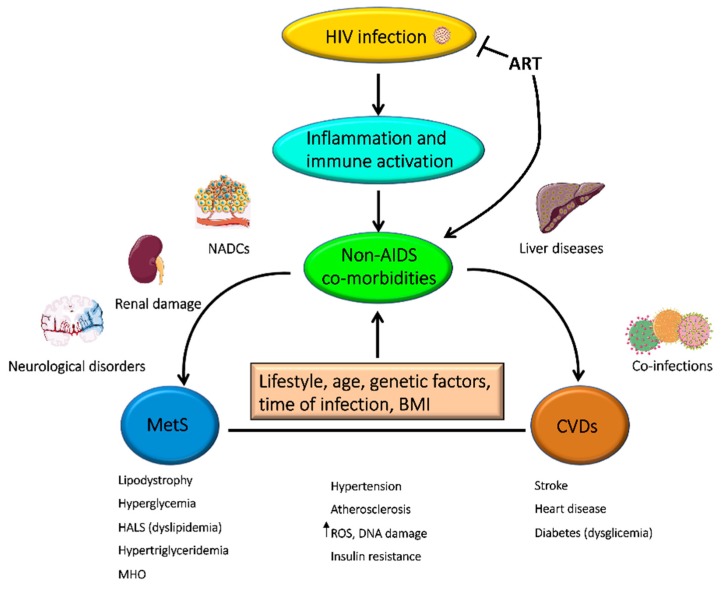
HIV causes persistent inflammation and chronic immune activation. Over the years and despite effective ART, this altered immunological status could lead to the development of non-AIDS co-morbidities in people living with HIV (PLWH). Many of these are identified as cardiovascular diseases (CVDs) or metabolic syndrome (MetS). NADCs: non-AIDS-defining cancers; HALS: HIV-associated lipodystrophy syndrome; MHO: metabolically healthy obesity; ROS: reactive oxygen species; BMI: body mass index. Images were obtained from Servier Medical Art images (http://smart.servier.com/).

The successful introduction of ART increased the life expectancy of PLWH and consequently their risk of developing non-AIDS co-morbidities, such as MetS and CVDs [78] (Figure 2).

The metabolic changes that occur in PLWH are initiated by the early impairment of the immune system caused by the virus, bringing deep changes in the cytokine network and causing persistent inflammation that is present despite ART initiation [79]. HIV infection results in oxidative stress and abnormal production of reactive oxygen species (ROS), causing endothelial dysfunction, tissue damage, chronic inflammation [80,81], and impairment of the autophagy mechanism [82]. Both HIV infection and ART treatment in HIV-infected mothers can also impact fetal metabolism by generating ROS that result in a higher production of peroxidized lipids and triglycerides in HIV-exposed infants compared with uninfected ones, leading them to develop a pro-inflammatory profile at birth [83].

MetS prevalence in PLWH is commonly evaluated by the Adult Treatment Panel III (ATP III) and International Diabetes Federation (IDF) criteria. Using these criteria, it was shown that the prevalence of MetS is comparable in PLWH versus HCs, but higher in PLWH under ART compared with ART-naïve PLWH [84,85]. Overall, patients with MetS were older, HIV-infected for a longer time, and presented with a higher body mass index (BMI). Meanwhile Rogalska-Płońska et al., analyzing a cohort of PLWH (with 64% HCV co-infection) and comparing the results with the general Polish population, found a higher prevalence of MetS in PLWH [86]. Some co-infections, such as HCV, have been associated with a higher incidence of MetS and CVDs that in PLWH may also increase their mortality rate [87].

PLWH, including perinatally HIV-infected (perHIV) patients, have a higher risk of developing cancer due to immunosuppression and co-infections with oncogenic viruses [88,89]. HIV may also cause cancer through a direct carcinogenic effect, the activation of pathways of inflammation, or ART toxicity [90]. Moreover, it has been shown that aberrant lymphangiogenesis, fundamental in the growth and survival of lymphomas, derives from a direct action of HIV proteins rather than the virus itself [91]. In PLWH, the Center for Disease Control and Prevention (CDC) classified malignancies as AIDS-defining cancers (ADCs) (including Burkitt lymphoma, Kaposi sarcoma, and cervical cancer) and all the others as non-AIDS-defining cancers (NADCs) [92]. NADC incidence in PLWH is two times higher than in the general population, despite the use of ART [93]. In addition, in the past few years, an increase in the number of NADCs has been recorded, while ADC cases are decreasing but still reported [94,95].

PLWH often develop nonalcoholic fatty liver disease [96]. A strong correlation was found between high levels of aldosterone (a steroid hormone that when dysregulated is associated with the development of CVDs) in PLWH with MetS and liver fat accumulation [97]. Obesity and type 2 diabetes mellitus (DM) are also associated with hepatic fibrosis and liver steatosis in PLWH with HIV mono-infection [98]. The combination of HIV infection with MetS seems to exacerbate the renal damage caused by the virus, with PLWH presenting a higher urinary albumin–creatinine ratio compared to non-HIV-infected patients with MetS [99]. Always related to renal damage, a correlation was found between levels of cystatin-C (a marker of kidney function recently shown to be associated with a risk of developing CVDs and DM) and MetS [100]. PLWH with MetS showed higher levels of aspartate transaminase, triglycerides, and blood pressure, and lower levels of cholesterol HDL compared to patients without MetS. The immune activation caused by HIV infection and ART and the increasing levels of circulating pro-inflammatory cytokines and LPS were shown to also be the cause of insulin resistance (IR), nowadays considered a chronic inflammatory disease in PLWH, and consequently a high concentration of blood glucose and the development of hyperinsulinemia and DM [101,102,103]. Chronic immune activation and high levels of pro-inflammatory cytokines in several tissues (liver, adipose tissue, gastrointestinal tract, muscle) are also associated with lipid alterations and IR [78]. Additionally, IR results in hypertension by altering the dynamics of vascular homeostasis [79]. Related to this, it was shown that LPS is also associated with hypertension in PLWH [104]. Xu et al. conducted a meta-analysis, concluding that hypertension in PLWH correlates with ART and older age and that it is linked to dysglycemia, hypertriglyceridemia, and lower levels of cholesterol HDL [105]. In another study where PLWH under ART, ART-naïve PLWH, and HCs were assessed for MetS, the authors did not find a prevalence of MetS or CVDs in any of the groups but found that triglycerides and total and LDL cholesterol were higher in PLWH under ART, while cholesterol HDL was higher in HCs [106]. The persistent status of inflammation and chronic immune activation are some of the causes of endothelial dysfunction, atherosclerosis and hypertension, which affect the functionality of the heart and the vascular system [107] and can be considered as a predictive factor of CVDs and cerebral vascular disease development in PLWH under ART. In fact, these individuals, compared with uninfected patients, present higher risk of stroke, particularly those of undetermined etiology, [108] and of neurological disorders (e.g., dementia, neurocognitive disorders, asymptomatic impairment) [109].

Untreated PLWH are at high risk of developing MetS not only because of HIV but also because their lifestyle (cigarette smoking, heavy alcohol use, physical activity, and poor diet), while PLWH under ART present higher risk of developing dyslipidemia and CVDs [8]. The same results were obtained in PLWH under ART who developed dyslipidemia, hypertension, and abnormalities in body fat distribution (lipodystrophy), compared to ART naïve individuals [110]. 

Together with MetS, another syndrome caused by both HIV and ART is HIV-associated lipodystrophy syndrome (HALS). HALS is a degenerative condition characterized by body fat redistribution and damage to the adipose tissue. It has been associated with an increased risk of developing MetS and CVDs [111] and is more diffused among women, making them a patient group with an elevated cardiovascular risk [112].

PLWH under ART with a diagnosis of HALS have a lower prevalence of obesity [113]. The prevalence of overweight and obesity in PLWH is the same as in the general population, although PLWH presenting anomalous levels of glucose and lipids face major challenges to maintain normal weight values and prevent IR [114]. Lake et al. showed that a condition called metabolically healthy obesity (MHO, a state of obesity that is not associated with any CVDs) is present in PLWH (AIDS, 2017) and to the same extent as in HCs, providing evidence that HIV infection and obesity are not correlated [115]. They also showed that non-obese PLWH were more likely than HCs to develop MetS. Furthermore, many studies have shown an association between hypertension and obesity in PLWH [116,117].

Assessment of risk factors in PLWH should be routinely carried out for prevention in clinic, as highlighted by Cibrián-Ponce et al., who showed that in a one-year period, the risk of developing CVDs increased in more than 50% of their cohort [118]. Moreover, in some studies it has been shown that HIV increases the risk of developing MetS and CVDs more in women than it does in men [85,119,120] and in perHIV more than in horizontally infected (horHIV) patients [121]. Additionally, genetic screening for metabolic complications caused by HIV infection has recently become part of clinical care as a predictive tool to identify PLWH at risk of developing MetS [122]. Individual genetic background screening for HLA-B^*^5701 has already been used in HIV clinical practice to identify PLWH at risk of developing abacavir (ABC) hypersensitivity [122,123].

## 5. Effects of HIV and ART Exposure on Inflammation, Immunodysfunction and Premature Aging in Perinatally HIV-Infected Children

HIV pathogenesis differs in perHIV compared with horHIV, due to age-related differences in the immune system at the time of infection, the route of transmission, and the timing of ART start. Indeed, all of the above-mentioned variables greatly impact immune system development in perHIV and the disease has a faster progression than horHIV in the absence of ART [124,125]. This is because the immune system of newborns is still immature and unable to mount an efficient immune response, resulting in high levels of HIV viremia, which decreases with the introduction of ART [126]. Despite the benefits achieved through effective early start of therapy, cumulative exposure to HIV or ART since and before birth can cause chronic immune activation and persistent inflammation in perHIV patients [127]. In this context, collaborative projects with the final aim of improving perHIV patient quality of life are currently under progress and will explore alternative strategies in order to reduce chronic immune activation and inflammation [74,128].

HIV infection, associated with high levels of pro-inflammatory cytokines, causes chronic immune activation, enhanced by HIV-induced gut disruption and dysbiosis [127,129]. Moreover, Persaud et al. has demonstrated that IL-6 correlates with intestinal fatty acid binding protein-2 (IFABP2), a biomarker of intestinal damage, suggesting an association between inflammation and intestinal integrity [130]. Indeed, gut immunity and alterations are not fully restored by effective ART, leading to microbial translocation. Recently, Sessa et al. showed that a distinct microbiota profile is correlated with high levels of sCD14 (a marker of microbial translocation), IL-6, ICAM-1, VCAM-1, and E-selectin (markers of endothelial activation) in perHIV. These data suggest that an altered microbiota profile could lead to inflammation and vascular activation, increasing the risk of developing non-AIDS co-morbidities, such as CVDs [131]. Such evidence provides the rationale for exploring specific probiotics as future interventions to reduce comorbidities experienced by perHIV patients. Nowadays, non-AIDS co-morbidities represent one of the major causes of morbidity and mortality in perHIV patients compared with age-matched HCs [132,133]. Indeed, perHIV patients present higher levels of glycemia, tryglicerides, CRP, TNF-α, systolic blood pressure, and carotid intima-media thickness compared with age-matched HCs, making them a group at higher risk of developing premature atherosclerosis, CVDs, and MetS [134].

Immune dysfunctions associated with HIV and ART exposure also include immunological exhaustion, as a result of a number of mechanisms impacting on both T and B cell compartments in perHIV patients. The immature immune system exposes perHIV patients to higher level of viral replication compared to adults, which in turn leads to higher expression of T cell surface activation markers such as CD38, HLA-DR, Ki67 [135], and apoptosis marker CD95 [136]. Moreover, dysfunctional immune-specific responses result in HIV-specific CD8 T cell exhaustion and apoptosis, characterized by the incremental loss of proliferative and effectors properties [137]. It has been shown that in perHIV, the percentage of activated CD8+CD38+ and exhausted CD8+PD1+ T cells is negatively associated with their telomere length, demonstrating the link between persistent immune activation and exhaustion with premature aging and immune senescence [59]. Overall, these findings together with alterations of memory T cells may also have an impact on the efficacy of childhood vaccination [138]. Related to this, we found that the process of immunosenescence and aging can also affect the B cell compartment, characterized by higher frequencies of mature activated B cells (CD19+CD10−CD21−) and double negative B cells (CD19+CD10−CD27−CD21−) in perHIV patients compared with their healthy counterparts and similarly to older healthy control [139,140,141]. B cell alterations have also been associated with a lower ability to respond to childhood vaccination [140,142]. Recently, we have also found that perturbation at a transcriptional level in the B cell compartment endures despite stable viral load in ART-treated perHIV patients [143]. All of these perturbations are only partially reversed by ART, and the immune reconstitution pattern mostly depends on age at ART start and thymus function. Immunological dysfunction and premature aging are of particular clinical relevance because they have been found to be associated with an elevated risk of developing cancer in perHIV patients [144]. To reduce this risk, it is crucial to pursue strategies of high compliance to ART, especially during adolescence, in order to induce long term viral control. In fact, it was recently shown that viremia, rather than exposure to ART, is positively related to premature aging of the immune system [59].

## 6. Novel ART Strategies to Reduce Inflammation: Two-Drug Therapies and Immunomodulators

In the absence of available treatments that can eradicate the virus completely, novel therapies are currently being explored in order to reduce ART-associated inflammation and the risk of developing non-AIDS co-morbidities. High expectations are currently laying on two-drug regimens. These therapies aim to minimize the toxicity and the elevated inflammation and immune activation associated with the use of nucleoside reverse transcriptase inhibitors (NRTIs) by reducing the number of NRTIs administered, assuming that this intervention would efficiently allow viral suppression. For example, the ANDES study showed similar virologic control in ART-naïve patients after 24 weeks of the darunavir booster ritonavir (DRV/R) + lamivudine (3TC) compared to standard triple ART with tenofovir disoproxil fumarate (TDF) [145]. The preliminary results were presented at the Conference on Retroviruses and Opportunistic Infections (CROI) in 2017, and the follow-up study is still ongoing. Furthermore, the ACTG A5353 study showed good early efficacy of dolutegravir (DTG, integrase strand transfer inhibitor, INSTI) + 3TC [146]. In the SWORD trial, patients were randomized to switch from triple NRTI-based therapy to once-daily oral DTG + rilpivirine (non-nucleoside reverse transcriptase inhibitors, NNRTI) [147]. This study demonstrated the non-inferiority of the dual therapy over triple regiments, with reduced NRTI-associated toxicity at 48 weeks after the switch. The same study also demonstrated that the dual regimen did not lead to an increase of inflammatory biomarkers or lipid concentration, but rather a deeper decrease in urine retinol-binding protein and urine β-2-microglobulin as compared with the triple regimen group. Of note, the choice of the NRTIs to be included should also be considered, especially with regards to MetS associated with inflammation. For example, in a study conducted on South African women, MetS prevalence was higher in those taking zidovudine as an NRTI than in those under TDF treatment [148]. Furthermore, the MASTER cohort showed that after 12 months of TDF and ABC reduction, levels of the platelet-to-lymphocytes ratio (PLR) decreased; given that high levels of PLR are associated with hypercholesterolemia and MetS, this study suggests that TDF and ABC reduction can reduce the risk of metabolic dysfunction [149]. In line with these studies, our and other groups also demonstrated that an extended use of TDF, especially in combination with protease inhibitor (PI), can negatively impact kidney function and osteoporosis [150,151]. In other studies, dual-drug or even single-drug (protease inhibitor, PI monotherapy) regimens were also introduced as a first-line therapy in adults, rather than used as simplification, as described above. For example, the PIVOT study showed the non-inferiority of PI monotherapy after three years of treatment [152,153]. Overall, it must be noted that all of these studies have the limitation of generally being extended for only one year or are still ongoing, thus providing the need for follow-ups to assess the concrete benefits of the study over long-term ART.

Beside ART regimen simplifications, the use of immunomodulators in order to reduce levels of immune activation and inflammation has also been discussed [154]. A very recent study by Soare et al. described the effects of the purinergic P2X receptor inhibitors [155]. P2X receptors are known to be expressed on CD4+ T cells, which are targets of HIV-1 infection; in addition, these proteins are known to be involved in the regulation of inflammatory pathways. The authors demonstrated that the P2X inhibitors NF449 and A438079 were not only able to impair HIV-1 infection, but surprisingly they induced the reduction of HIV-1-stimulated IL-10 and IL-1β production in human tonsil cells. Thus, this drug class is quite promising for this dual effect. Another recent example is provided by the statins: this drug class exhibits immunomodulatory effects through different mechanisms including cytokine production, expression of HLA class II antigens on macrophages, and inhibition of the expression of co-stimulatory molecules such as CD40, CD8, CD86, and antigen-presenting cells. Numerous studies have shown that atorvastatin, rosuvastatin, or pravastatin formulations render statins compatible for co-administration with ART, with rosuvastatin not being recommended with PI regimens [156]. All drugs showed efficacy in reducing levels of inflammatory markers. Furthermore, one recent study confirms previous evidence showing that atorvastatin also supports the reduction of T cell immune activation levels and exhaustion [157]. An ongoing study, the REPRIEVE trial, is testing the effect of pitavastatin, a new class of statin that did not show important adverse reactions to ART, in the prevention of CVDs in PLWH. The goal of this study is to show that this class of drugs, by decreasing cholesterol levels in circulation, will be able to lower inflammation and immune activation [158]. Another novel therapeutic strategy aiming at reducing inflammation in PLWH is based on the use of canakinumab, a human monoclonal anti-IL-1β antibody already approved by the FDA for CVD risk reduction. As shown by Hsue et al., a single dose of canakinumab was able to significantly reduce levels of IL-6, CRP, and sCD163 at eight weeks after administration in a small group of PLWH [159]. Furthermore, the sevelamer carbonate, a phosphate-lowering drug, has been showed to decrease levels of inflammatory markers. Navarro–Gonzalez et al. showed that this drug reduced levels of circulating LPS, sCD14, IL-6, and CRP in ART-treated patients [160]. Different effects were observed by Sandler et al. on ART-naïve patients: his data suggested that sevelamer was inefficient in reducing levels of LPS or sCD14, whereas it was associated with reduction of LDL [161]. Other immunomodulators are chloroquine and its analogue hydroxychloroquine. These drugs are generally administrated during tuberculosis infection, but they have been proven to impair HIV replication and functionality by targeting gp120 protein production, by reducing levels of intracellular iron that is required to support viral replication, and by impacting on Tat and integrase functionality [162]. Moreover, chloroquine enabled good CD4 recovery in immune non-responding HIV-infected patients under ART [163]. The 5–10 year view in this field is expected to be enriched with many other data aiming to show the benefits of immunology-friendly drugs to enhance ART.

## 7. Conclusions

HIV infection leads to persistent inflammation, chronic immune activation, thymic dysfunction, gut microbial translocation, and ultimately, premature aging. The increased production of pro-inflammatory cytokines and LPS, dysfunctional Treg production in the thymus, immunosenescence, ART toxicity, and co-infections may lead PLWH to develop non-AIDS co-morbidities, such as MetS, CVDs, and NADCs. New therapy approaches aiming at minimizing the effect of immunological dysfunction and avoiding drug toxicity are currently under study. The management of these co-morbidities is challenging. Routine metabolic screenings should become an essential part of routine HIV care. In addition, measuring personal patients’ risks at every visit, starting immediately from the time of HIV diagnosis, will help to improve PLWH’s quality life.
